# Pumilio RNA-Binding Family Member 1 Plays a Promoting Role on Pancreatic Cancer Angiogenesis

**DOI:** 10.1155/2022/9202531

**Published:** 2022-12-14

**Authors:** Haisu Dai, Yan Jiang, Zhipeng Liu, Xingxing Su, Yishi Yang, Zhiyu Chen

**Affiliations:** Department of Hepatobiliary Surgery, First Affiliated Hospital, Army Medical University, Chongqing 400038, China

## Abstract

Our previous studies showed that Pumilio RNA-binding family member 1 (PUM1) gene is abnormally expressed in pancreatic cancer (PC) tissues, and its knockdown suppresses the growth and metastasis of PC cells. Here, we aimed to further investigate its role in angiogenesis. Immunohistochemical assays were carried out to analyze CD31 and PUM1 expression levels in PC tissues and in subcutaneous xenograft tumors. CD31 levels in PC tissues are expressed as microvessel density (MVD). MVD value was positively correlated with PUM1 protein expression. PUM1 was successfully overexpressed or silenced in the PC cell lines. The proliferation, migration, invasion, and tube formation ability of HUVECs were enhanced when cocultured with PC cells overexpressing PUM1. PUM1 overexpression increased extracellular and intracellular VEGFA protein levels in PC cells. Moreover, angiogenesis-related signaling in HUVECs was activated when HUVECs were cocultured with PC cells overexpressing PUM1. Nevertheless, PC cells silenced with PUM1 had the opposite effect. Moreover, subcutaneous xenograft tumors overexpressing PUM1 have the higher expression level of CD31, while subcutaneous xenograft tumors silencing PUM1 have the lower expression level of CD31. In conclusion, PUM1 in PC cells may play a promoting role in PC angiogenesis. PUM1 may be a new regulator of angiogenesis in PC cells.

## 1. Introduction

Pancreatic cancer (PC) is a highly lethal malignancy [1]. It ranks second in the cause of death of malignancies in the digestive system and the fourth most lethal malignancy in both males and females in the United States [2]. The main treatment of PC includes surgical resection, chemoradiotherapy, and targeted therapy. The 5-year survival rate of PC patients is less than 10% [3, 4]. The early clinical symptoms of primary PC are not typical, so a large proportion of patients present at an advanced stage. In addition, the diagnosis of rare tumors caused by ectopic pancreatic tissue is challenging [5]. The preoperative diagnosis of the malignant transformation of ectopic pancreatic tissue is often difficult with conventional imaging, and biopsy is the most common method of diagnosis [5]. Immunoglobulin G4-related disease (IgG4-RD) may involve one or multiple organs [6]. IgG4-RD involving the pancreas may be misdiagnosed as pancreatic cancer (pancreatic head cancer or bile duct carcinoma) [6]. Therefore, the early and accurate diagnosis of PC is important for the prognosis of patients. In addition, neuropathic pain caused by PC seriously affects the quality of life of patients, negatively affects the prognosis of patients, and leads to increased psychological stress [7]. Neuropathic pain associated with PC remains undertreated [7]. Therefore, it is significant to understand the molecular mechanism of PC metastasis and explore the therapeutic targets of PC at an advanced stage for finding new treatments.

The growth and metastasis of malignant solid tumors are closely related to the blood vessels in the tumor area [8]. The new capillaries in the tumor area are the material basis for tumor growth and metastasis. PC is a solid tumor. Several studies have demonstrated that angiogenesis is closely related to the tumor growth and metastasis of PC [3, 9]. One important mechanism in PC is that tumor cells induce the surrounding tissues to produce proangiogenic factors to promote angiogenesis and provide a suitable microenvironment for the tumor growth and metastasis [8]. Therefore, it is important to identify new angiogenesis regulators in PC cells and understand their mechanism, which has important theoretical significance and clinical practical value for antiangiogenesis therapy of tumor vessels.

In our previous studies, we found that Pumilio RNA-binding family member 1 (PUM1), a sequence-specificRNA-binding protein, is abnormally expressed in PC tissues, and its knockdown suppresses cell growth and metastasis of PC cells [10], indicating that PUM1 may play an oncogene in the pathological process of PC. Other researchers also reported that PUM1 functions as an oncogene in ovarian cancer, colon cancer, and nonsmall cell lung cancer [11–13]. PUM1 can regulate protein translation by binding to the 3′ end of the messenger RNA [14–16]. Besides its role in cancers, PUM1 is also involved in stem cell fate and neurological function [17].

Here, we aimed to investigate the role of PUM1 in angiogenesis. First, we analyzed the expression correlation between PUM1 and the neovascular marker CD31 in PC tissues. Second, we evaluated the effect of PC cells overexpressing or silencing PUM1 on the function of vascular endothelial cells by constructing a Tcoculture system of PC cells and human umbilical vein endothelial cells (HUVECs).

## 2. Materials and Methods

### 2.1. Correlation Analysis of PUM1 Level and Microvessel Density (MVD) Values

Forty-eight patients diagnosed as having PC in our hospital were enrolled. PC tissues were collected during surgery. The study about PC tissues was approved by the research ethics committee of our hospital (No. KY2020138) in accordance with the Helsinki Declaration. Written informed consent was obtained from all the patients. PC tissue microarrays were prepared as previously described [10]. The primary antibody of CD31, an angiogenesis marker, was used to detect the distribution of microvasculature. Immunohistochemical (IHC) assays were performed by Shanghai Outdo Biotech Co., Ltd. Anti-CD31 antibody was purchased from Thermo Fisher Scientific (Waltham, MA, USA), and its dilution was 1 : 800. The staining result for CD31 was expressed by microvessel density (MVD) [18]. PUM1 levels in PC tissue microarrays were analyzed as previously described [10]. The potential effect of PUM1 on angiogenesis was evaluated by the correlation between PUM1 levels and MVD values.

### 2.2. Construction of PUM1 Stable Overexpression or Knockdown Cells

The construction of PUM1 stable knockdown MIA PaCa-2 and PANC-1 cells has been described in our previous report [10]. PUM1 stable knockdown PC cells were named as sh-PUM1 and their negative control sh-NC. Lentivirus expressing the full-length open reading frame of PUM1 (NM_001020658) was provided by GenePharma (Shanghai, China) and named as lv-PUM1. To generate PC cells stably overexpressing PUM1, lv-PUM1 was used to infect MIA PaCa-2 and PANC-1 cells. The cell lines stably overexpressing PUM1 were named ov-PUM1 and their negative control ov-NC.

### 2.3. VEGFA Level Measurements Using Enzyme-Linked Immunosorbent Assay (ELISA)

VEGFA protein levels in the culture medium of sh-NC, sh-PUM1, ov-NC, and ov-PUM1 groups were measured using VEGF Human ELISA Kit (Thermo Fisher Scientific).

### 2.4. Measurement of HUVECs Proliferation

Transwell insert with 96-well was used. HUVECs (1 × 10^5^ cells in 600 *μ*L culture medium) were cultured overnight in the lower chamber. The next day, cells of sh-NC, sh-PUM1, ov-NC, and ov-PUM1 groups were, respectively, cultured in the upper chamber. After coculturing for 0, 24, and 48 h, the absorbance (OD value) at 450 nm wavelength was measured according to the instruction of Cell Counting Kit-8.

### 2.5. EdU Assay

HUVECs (1 × 10^5^ cells in 600 *μ*L of culture medium) were cultured overnight in the lower chamber of 96-well Transwell inserts. The next day, cells of sh-NC, sh-PUM1, ov-NC, and ov-PUM1 groups were cultured in the upper chamber. At the same time, to label cells with EdU, 100 *μ*L of cell culture medium of HUVECs was replaced with 100 *μ*L of 2 x EdU solution (Abcam, Cambridge, MA, USA). After culturing for 4 h, HUVECs were fixed and permeabilized and then incubated with EdU reaction solution (Abcam) for 30 min at 22 ± 2°C in the dark. After washing once with PBS, HUVECs in the lower chamber were stained with Hoechst 33342 to stain the cell nuclei. Finally, the cells were visualized under a fluorescence microscope. The percentage of EdU-positive cells to total cells was calculated as the following formula: percentage of EdU-positive cells = (number of EdU-positive cells/number of Hoechst 33342-stained cells) × 100% [19].

### 2.6. Wound Healing Assay

Before cell culture, three straight lines were drawn on the back of the bottom of the Transwell chamber with a marker. HUVECs cells (4 × 10^5^ cells in 600 *μ*L culture medium) were cultured overnight in the lower chamber to form a single cell layer. The next day, a sterile pipette tip (10 *µ*L) was used to scratch cells perpendicular to the straight line as far as possible, and scratched cells were washed off. At the same time, cells of each group were cultured in the upper chamber. The wound healing condition of HUVECs cells was observed, and five images were randomly captured at 0 and 24 h after scratching. Through quantifying the wound area at 0 and 24 h after scratching, the percentage of wound healing was calculated [20].

### 2.7. Matrigel-Based Tube Formation Assay

HUVECs cells (1 × 10^5^) were cultured in a 24-well Transwell insert precoated with Matrigel (BD Biosciences, Bedford, MA). MIA PaCa-2 and PANC-1 cells of sh-NC, sh-PUM1, ov-NC, and ov-PUM1 groups were cultured in the upper chamber. After coculturing for 6 h, HUVECs cells were observed and the branch node number was counted. The average number was used as the value of each group.

### 2.8. Transwell Migration and Transwell-Matrigel Invasion Assays

To measure the migration ability, HUVECs (1 × 10^4^ cells in 200 *μ*L culture medium) were cultured in the upper chamber of a 24-well Transwell insert. Cells of sh-NC, sh-PUM1, ov-NC, and ov-PUM1 groups were cultured in the lower chamber. After coculturing for 24 h, the upper chamber was washed with PBS. Then, the upper chamber was put in a beaker with 5% glutaraldehyde to fix at 4°C. After fixing for 20–30 min, the upper chamber was stained with crystal violet (0.5%) for 5–10 min. After washing twice with PBS, the nonmigrated cells were removed using a cotton swab. For the Transwell-Matrigel invasion assay, HUVECs (1 × 10^4^ cells in 200 *μ*L culture medium) were cultured in the upper chamber precoated with Matrigel (Sigma-Aldrich, St Louis, MO, USA), and the protocol was the same as the Transwell migration assay. Five microscopic fields were randomly observed under the microscope. The average was used as the number of migratory or invaded HUVECs cells.

### 2.9. Western Blotting

HUVECs (4 × 10^5^ cells in 2 mL culture medium) were cultured in the lower chamber 6-well Transwell inserts. Cells of sh-NC, sh-PUM1, ov-NC, and ov-PUM1 groups were cultured in the upper chamber. After coculturing at 37°C in 5% CO_2_ incubator for 24 h, HUVECs were harvested to detect the protein level of VEGFR2, phosphorylated MEK1 (p-MEK1), MEK1, phosphorylated ERK1 (p-ERK1), ERK1, Notch1, and DLL4. The western blotting protocol was the same as that described in our previous study [10]. The dilution ratio and catalogue number of primary antibodies were as follows: PUM1 (1 : 2000; ab92545, Abcam, Cambridge, UK), VEGFA (1 : 500; ab51745, Abcam), VEGFR2 (1 : 800; ab39638, Abcam), p-MEK1 (1 : 1500; orb544327, Biorbyt, Cambridge, UK), MEK1 (1 : 1000; ab109556, Abcam), p-ERK1 (1 : 3000; orb10606, Biorbyt), ERK1 (1 : 3000; ab78918, Abcam), NOTCH1 (1 : 1000; ab167441, Abcam), and DLL4 (1 : 800; ab176876, Abcam).

### 2.10. Measurement of PUM1 and CD31 in Subcutaneous Xenograft Tumors

Subcutaneous xenograft tumor models were constructed as previously described [10]. Twenty-four athymic nude mice were divided into eight groups (*n* = 3) and were subcutaneously injected in the right armpit region with MIA PaCa-2 and PANC-1 cells of sh-NC, sh-PUM1, ov-NC, and ov-PUM1 groups (4 × 10^6^ cells in 0.2 mL of PBS). Twenty-eight days after the injection, subcutaneous xenograft tumor tissues were isolated and fixed in 4% paraformaldehyde. PUM1 and CD3 levels in subcutaneous xenograft tumors were measured by immunohistochemical analysis using the same method mentioned above.

### 2.11. Statistical Analysis

Statistical analysis was performed by using GraphPad Prism software version 7.0 (GraphPad Software Inc., San Diego, CA). Statistical significance was set at *p* < 0.05. Data are expressed as mean ± standard deviation. Statistical differences between the two groups were analyzed using *t*-tests. The correlation between MVD and PUM1 levels in PC tissues was analyzed by linear regression analysis.

## 3. Results

### 3.1. PUM1 and CD31 Expression in PC Tissues

**Figure 1:**
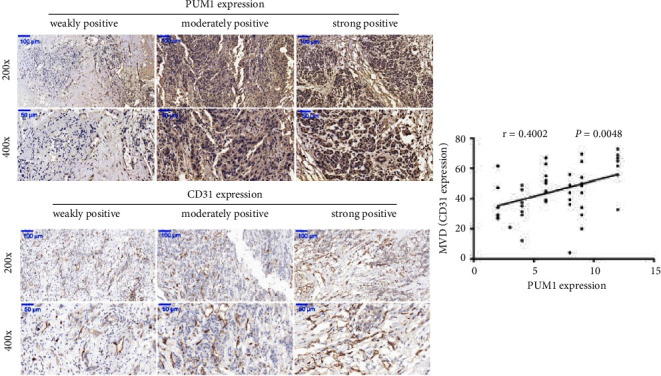
Positive correlation between PUM1 and CD31 expression in pancreatic cancer (PC) tissues. Immunohistochemical (IHC) assays were performed to measure PUM1 and CD31 expressions in PC tissues (*n* = 48). Left panels: representative images of IHC. Right panel: correlation between PUM1 and CD31 levels. CD level was expressed as microvessel density (MVD).

To explore the role of PUM1 in regulating angiogenesis, we first analyzed the correlation between PUM1 and CD31 expression in forty-eight PC tissues. The CD31 level was expressed as the MVD. As shown in Figure 1, the MVD value was positively correlated with PUM1 protein expression (*r* = 0.4002, *p* = 0.0048).

### 3.2. Effect of PUM1 Overexpression or Silencing on VEGFA Expression in PC Cell Lines

**Figure 2:**
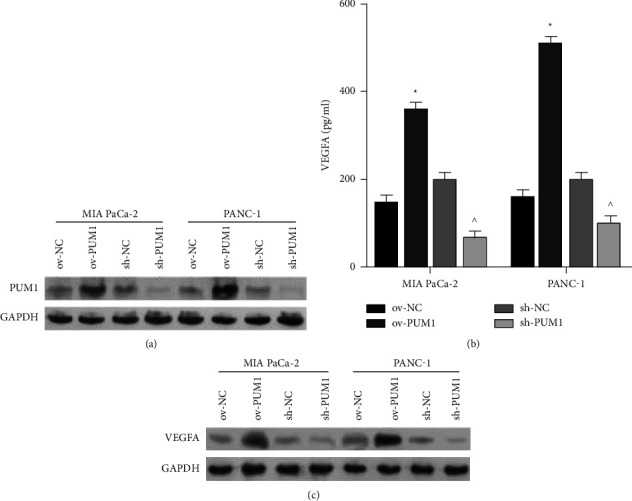
VEGFA expression in pancreatic cancer cells overexpressed or silenced PUM1. PUM1 protein levels in these cells were detected using Western blotting (a). VEGFA levels in these cells were detected using ELISA assay (b) and Western blotting (c) ^∗^*p* *<* 0.05, ov-PUM1 vs. ov-NC; ^ *p* < 0.05, sh-PUM1*vs*. sh-NC.

As shown in Figure 2 A, the PUM1 protein level was increased in the ov-PUM1 compared to the ov-NC and decreased in the sh-PUM1 compared to the sh-NC, indicating that PUM1 was successfully overexpressed or silenced in the PC cell lines.

We measured extracellular and intracellular VEGFA protein levels in PC cells. The VEGFA protein level was increased in the ov-PUM1 compared to the ov-NC and decreased in the sh-PUM1 compared to the sh-NC (Figures 2(b) and 2(c)), indicating that PUM1 overexpression can promote VEGFA protein secretion in PC cells, while silencing PUM1 has the opposite effect.

### 3.3. Effect of PC Cells Overexpressing or Silencing PUM1 on Proliferation of HUVECs

**Figure 3:**
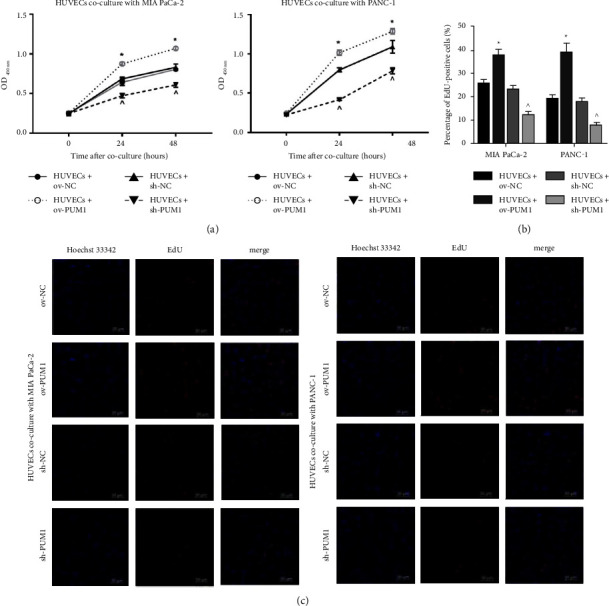
Effect of pancreatic cancer cells overexpressed or silenced PUM1 on proliferation of HUVECs. To construct a transwell co-culture system, MIA PaCa-2 and PANC-1 cells of ov-NC, ov-PUM1, sh-NC, and sh-PUM1 were cultured in the upper chamber of a transwell insert, and HUVECs were cultured in the lower chamber. (a) Absorbance (OD value) at 450 nm wavelength was detected by cell proliferation assay to reflect the proliferation of HUVECs. (b)–(c): Percentage of EdU-positive cells was analyzed by EdU assay to reflect the proliferation of HUVECs. Panel B is the quantitative statistical results. Panel C is the representative image of each group. ^∗^*p* < 0.05, ov-PUM1 vs. ov-NC; ^*p*  < 0.05, sh-PUM1 vs. sh-NC.

To explore the function of PUM1 in angiogenesis, a Transwell coculture system of PC cells and HUVECs was constructed to analyze the effect on the proliferation of HUVECs. The OD value at 450 nm and percentage of EdU-positive cells were higher in the HUVECs + ov-PUM1 than that in the HUVECs + ov-NC and were lower in the HUVECs + sh-PUM1 than that in the HUVECs + sh-NC (Figures 3(a)–3(c)). These results revealed that PC cells overexpressing PUM1 could promote the proliferation of HUVECs, while silencing PUM1 in PC cells has the opposite effect.

### 3.4. Effect of PC Cells Overexpressing or Silencing PUM1 on Migration and Invasion of HUVECs

**Figure 4:**
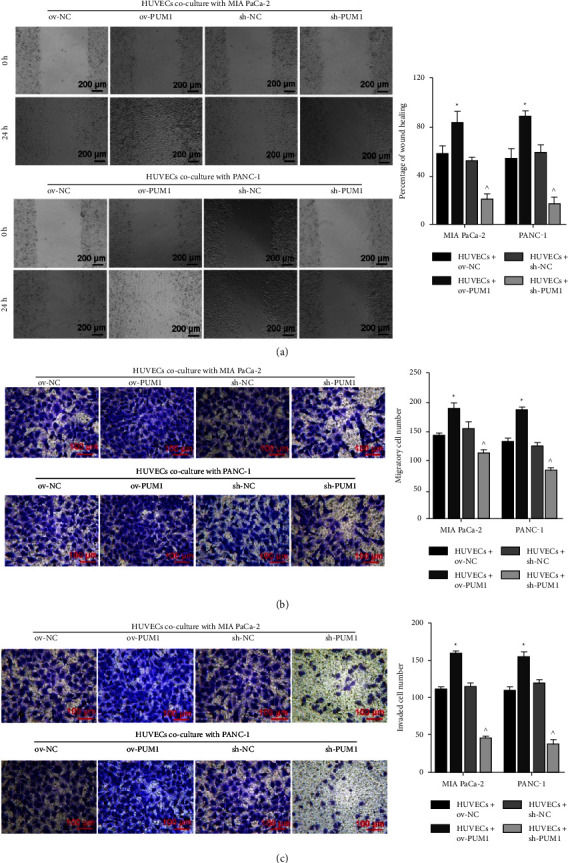
Effect of pancreatic cancer cells overexpressed or silenced PUM1 on migration and invasion of HUVECs. To construct a transwell co-culture system of wound healing assay (a), MIA PaCa-2 and PANC-1 cells of ov-NC, ov-PUM1, sh-NC, and sh-PUM1 were cultured in the upper chamber of a transwell insert, and HUVECs were cultured in the lower chamber. For Transwell migration (b) and transwell-Matrigel invasion (c) assay, MIA PaCa-2 and PANC-1 cells of sh-NC, sh-PUM1, ov-NC, ov-PUM1 group were cultured in the lower chamber of a transwell insert, and HUVECs were cultured in the upper chamber. For panels A, B, and C left is representative images, and right is the statistical result of three independent experiments. ^∗^*p* < 0.05, ov-PUM1 vs. ov-NC; ^ *p* < 0.05, sh-PUM1*vs*. sh-NC.

To analyze the effect of PUM1 on migration and invasion of HUVECs, a Transwell coculture system of PC cells and HUVECs was constructed. The percentage of wound healing, migratory cell number, and invaded cell number were higher in HUVECs + ov-PUM1 than that in the HUVECs + ov-NC and were lower in the HUVECs + sh-PUM1 than that in the HUVECs + sh-NC (Figures 4(a)–4(c)). These results suggest that PC cells overexpressing PUM1 can promote the migration and invasion of HUVECs, while silencing PUM1 in PC cells has the opposite effect.

### 3.5. Effect of PC Cells Overexpressing or Silencing PUM1 on Tube Formation Ability of HUVECs

**Figure 5:**
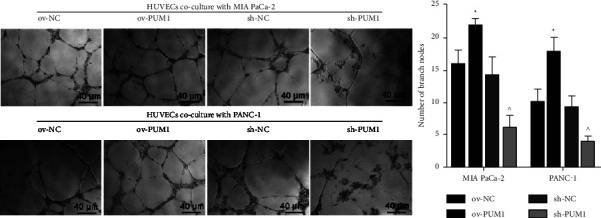
Effect of pancreatic cancer cells overexpressing or silencing PUM1 on tube formation ability of HUVECs. To construct a transwell co-culture system, MIA PaCa-2 and PANC-1 cells stably overexpressing PUM1 (ov-PUM1), silencing PUM1 (sh-PUM1), and their negative control cells (ov-NC and sh-NC) were cultured in the upper chamber of a transwell insert, and HUVECs were cultured in the lower chamber. Matrigel-based tube formation assay was performed to assess tube formation ability of HUVECs. Left panel is representative images, and right panel is the statistical result of three independent experiments. ^∗^*p* < 0.05, ov-PUM1 vs. ov-NC; ^ *p* < 0.05, sh-PUM1 vs. sh-NC.

To further explore the function of PUM1 in angiogenesis, we constructed a Transwell coculture system of PC cells and HUVECs to analyze the effect on tube formation ability of HUVECs. The number of branch nodes was higher in the HUVECs + ov-PUM1 than in the HUVECs + ov-NC and was lower in HUVECs + sh-PUM1 than that in the HUVECs + sh-NC (Figure 5). These results suggest that PC cells overexpressing PUM1 can enhance the tube formation ability of HUVECs, while silencing PUM1 in PC cells has the opposite effect.

### 3.6. Effect of PC Cells Overexpressing or Silencing PUM1 on Angiogenesis-Related Signaling of HUVECs

**Figure 6:**
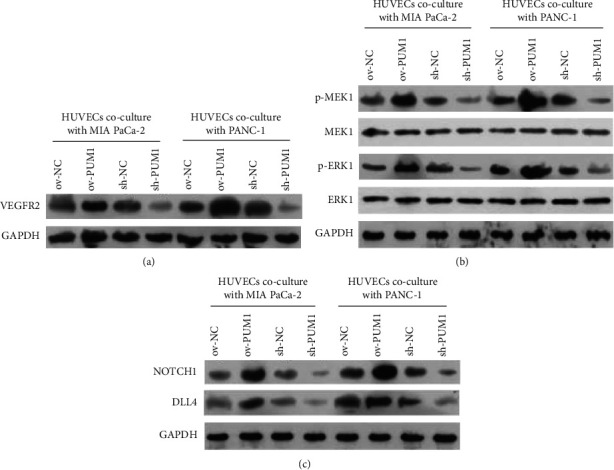
Effect of pancreatic cancer cells overexpressed or silenced PUM1 on angiogenesis-related signaling of HUVECs. To construct a transwell co-culture system, MIA PaCa-2 and PANC-1 cells of ov-NC, ov-PUM1, sh-NC, and sh-PUM1 were cultured in the upper chamber of a transwell insert, and HUVECs were cultured in the lower chamber. Protein levels in HUVECs were detected by Western blot.

To further explore the function of PUM1 in angiogenesis, a Transwell coculture system of PC cells and HUVECs was constructed, and the protein levels of angiogenesis-related signaling in HUVECs were detected. VEGFR2, p-MEK1, p-ERK1, NOTCH1, and DLL4 levels were higher in the HUVECs + ov-PUM1 than in the HUVECs + ov-NC and were lower in HUVECs + sh-PUM1 than that in the HUVECs + sh-NC (Figures 6(a)–6(c)). PUM1 had no effect on total levels of MEK1 and ERK1 (Figure 6(b)). These results suggest that PC cells overexpressing PUM1 can activate angiogenesis-related signaling in HUVECs, while silencing PUM1 in PC cells has the opposite effect. In addition, the effect of the coculture time on angiogenesis-related signaling of HUVECs was also analyzed. After 12 h of coculturing, MIA PACA-2 cells overexpressing PUM1 could obviously activate the angiogenesis-related signaling in HUVECs, and this effect lasted for at least 72 h (Figure S1). The activation peaked at 24 h (Figure S1).

### 3.7. Correlation between PUM1 and CD31 Expression in Subcutaneous Xenograft Tumors

**Figure 7:**
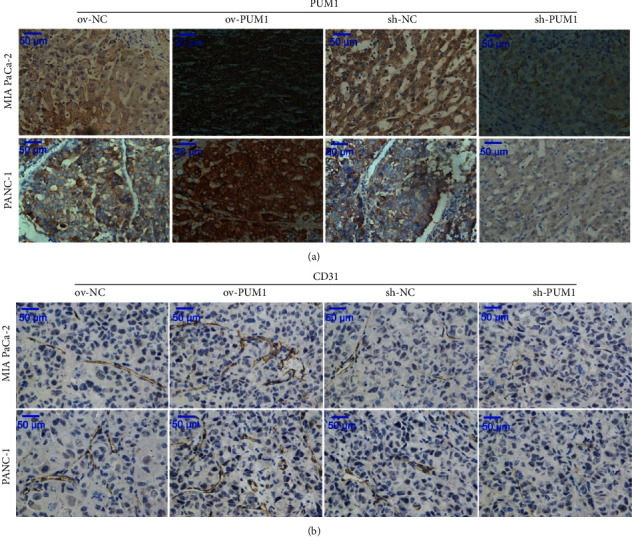
Expression levels of PUM1 and CD31 in subcutaneous xenograft tumors. Subcutaneous xenograft tumor models were constructed using MIA PaCa-2 and PANC-1 cells stably overexpressing PUM1 (ov-PUM1), silencing PUM1 (sh-PUM1), and their negative control cells (ov-NC and sh-NC). PUM1 (a) and CD31 (b) in subcutaneous xenograft tumors were measured using immunohistochemical analysis.

To further confirm the function of PUM1 in regulating angiogenesis, we measured PUM1 and CD31 levels in subcutaneous xenograft tumors generated using PC cell lines overexpressing or silencing PUM1. Both PUM1 and CD31 protein levels were increased in the subcutaneous xenograft tumors of ov-PUM1 compared to the ov-NC and decreased in the subcutaneous xenograft tumors of sh-PUM1 compared to the sh-NC (Figures 7(a) and 7(b)). These results suggest that subcutaneous xenograft tumors overexpressing PUM1 have higher expression level of CD31, while subcutaneous xenograft tumors silencing PUM1 have lower expression level of CD31.

## 4. Discussion

We discussed the role of PUM1 in angiogenesis during the pathological process of PC for the first time. This study will lay a theoretical foundation for the research and development of targeted drugs for antiangiogenesis therapy of PC.

We found that CD31 protein levels (expressed as MVD) were positively correlated with the protein expression of PUM1 in PC tissues. In addition, subcutaneous xenograft tumors overexpressing PUM1 have higher expression level of CD31, while subcutaneous xenograft tumors silencing PUM1 have lower expression level of CD31. CD31, officially named platelet and endothelial cell adhesion molecule 1, is an endothelial cell surface marker [21, 22]. In the field of angiogenesis, CD31 is widely used as a vascular marker [21, 22]. Therefore, our results revealed that the PUM1 level is positively correlated with the number of blood vessels in PC tissues. We hypothesized that PUM1 may participate in angiogenesis and may be a new angiogenesis regulator in PC cells.

To verify our hypothesis, PC cells overexpressing and silencing PUM1 were constructed and their effect on angiogenic features of HUVECs was analyzed *in vitro* [23]. Our results showed that the angiogenic features of HUVECs, including the abilities of proliferation, migration, invasion, and tube formation, were enhanced when cocultured with PC cells overexpressing PUM1. Angiogenesis is an extremely complex process. Furthermore, the proliferation, migration, and invasion of vascular endothelial cells are important processes of angiogenesis [24]. The next crucial step is endothelial cell budding and capillary network formation [24]. Matrigel-based tube formation assay can simulate the process of endothelial cell budding and capillary network formation. These results suggest that PC cells overexpressing PUM1 can promote angiogenesis. The activation of angiogenesis-related signaling in HUVECs supported that PC cells overexpressing PUM1 can promote angiogenesis. This conclusion is also supported by the suppressive effect of PC cells that silenced PUM1 on angiogenic features of HUVECs.

Moreover, PUM1 overexpression promoted VEGFA protein secretion in PC cells. VEGFA is a highly specific provascular endothelial cell growth factor (VEGF). Tumor angiogenesis is mainly dependent on VEGFA-driven responses [25]. Therefore, PUM1 may play a role in PC angiogenesis by promoting VEGFA secretion in PC cells. PUM1 is a RNA-binding protein [26]. PUM1 promotes degradation and/or translational repression of its target mRNAs [27, 28]. Due to PUM1 overexpression promoted VEGFA expression, so we predicated that VEGFA mRNA is not the target of PUM1. In the future, we will explore the molecular mechanism underlying PUM1 upregulation of VEGFA protein levels in PC cells.

We also found that PC cells overexpressing PUM1 could increase the protein level of VEGFR2 in HUVECs. VEGFR2 is one receptor of VEGFA. VEGFA binding to VEGFR2 can activate many angiogenesis-related signaling, such as MEK/ERK signaling pathway [29, 30]. Our results also suggest that the MEK/ERK signaling pathway in HUVECs can be activated by PC cells overexpressing PUM1. Based on these results, we hypothesize that PUM1 plays its promoting role on angiogenesis through VEGFA/VEGFR2/MEK/ERK signal transduction pathway. This hypothesis needs more evidences to confirm. In addition, other angiogenesis-related signaling may also be involved in the regulation of PUM1 on angiogenesis.

Another issue is how PUM1 regulates VEGFR2 expression. It is reported that VEGFR2 transcription can be regulated by several transcription factors that in turn are regulated by numerous signaling, such as Notch signals [31, 32]. Therefore, we hypothesize that PUM1 promotes VEGFR2 expression through DLL/NOTCH pathway in HUVECs. In the future, we will explore the molecular mechanism underlying PUM1 activation of DLL/NOTCH pathway in HUVECs.

In recent years, increasing attention has been paid to the role of vegetables, such as Allium, in the prevention and treatment of cancer [33]. Active constituents derived from Allium have anti-inflammatory, antioxidant, antimicrobial, and anticancer properties [33]. Remarkably, experimental results demonstrate that Allium extracts have the potential to inhibit angiogenesis [33]. Therefore, PUM1 may be the target of Allium extracts in regulating angiogenesis. Elucidation of their relationship will provide insights into the molecular mechanism of Allium extracts and expand the application of PUM1-targeted therapeutic strategies.

In conclusion, the expression level of PUM1 is positively correlated with the number of blood vessels in PC tissues and subcutaneous xenograft tumors, and *in vitro* assays showed that PC cells overexpressing PUM1 promoted cell proliferation, migration ability, invasion ability, tube formation ability, and angiogenesis-related signaling in HUVECs. Our results suggest that PUM1 plays a promoting role in PC angiogenesis, and it may be a new target for anti-angiogenesis therapy in PC.

## Data Availability

The datasets used and/or analyzed during the current study are available from the corresponding author upon reasonable request.

## Abbreviations

PC: Pancreatic cancer
MVD: Microvessel density (MVD)
NC: Negative control
PUM1: Pumilio RNA-binding family member 1
sh-PUM1: PUM1 shRNA
HUVECs: Human umbilical vein endothelial cells.

## Supplementary Materials

Supplementary Materials
*Figure S1*. Effect of pancreatic cancer cells overexpressed PUM1 on angiogenesis-related signaling of HUVECs at different time-points. To construct a Transwell coculture system, MIA PaCa-2 cells stably overexpressing PUM1 (ov-PUM1) and negative control cells (ov-NC) were cultured in the upper chamber, and HUVECs were cultured in the lower chamber. After coculturing for different time, western blot was performed to detect protein levels.
