# Supplementary material for: Pumilio RNA-Binding Family Member 1 Plays a Promoting Role on Pancreatic Cancer Angiogenesis
Source: Can J Gastroenterol Hepatol. 2022 Dec 14;2022:9202531. doi: 10.1155/2022/9202531 (PMC11410436; doi:10.1155/2022/9202531)
Supplement: Supplementary Materials — Figure S1. Effect of pancreatic cancer cells overexpressed PUM1 on angiogenesis-related signaling of HUVECs at different time-points. To construct a Transwell coculture system, MIA PaCa-2 cells stably overexpressing PUM1 (ov-PUM1) and negative control cells (ov-NC) were cultured in the upper chamber, and HUVECs were cultured in the lower chamber. After coculturing for different time, western blot was performed to detect protein levels. [file 9202531.f1.docx]

**Supplementary file**

**
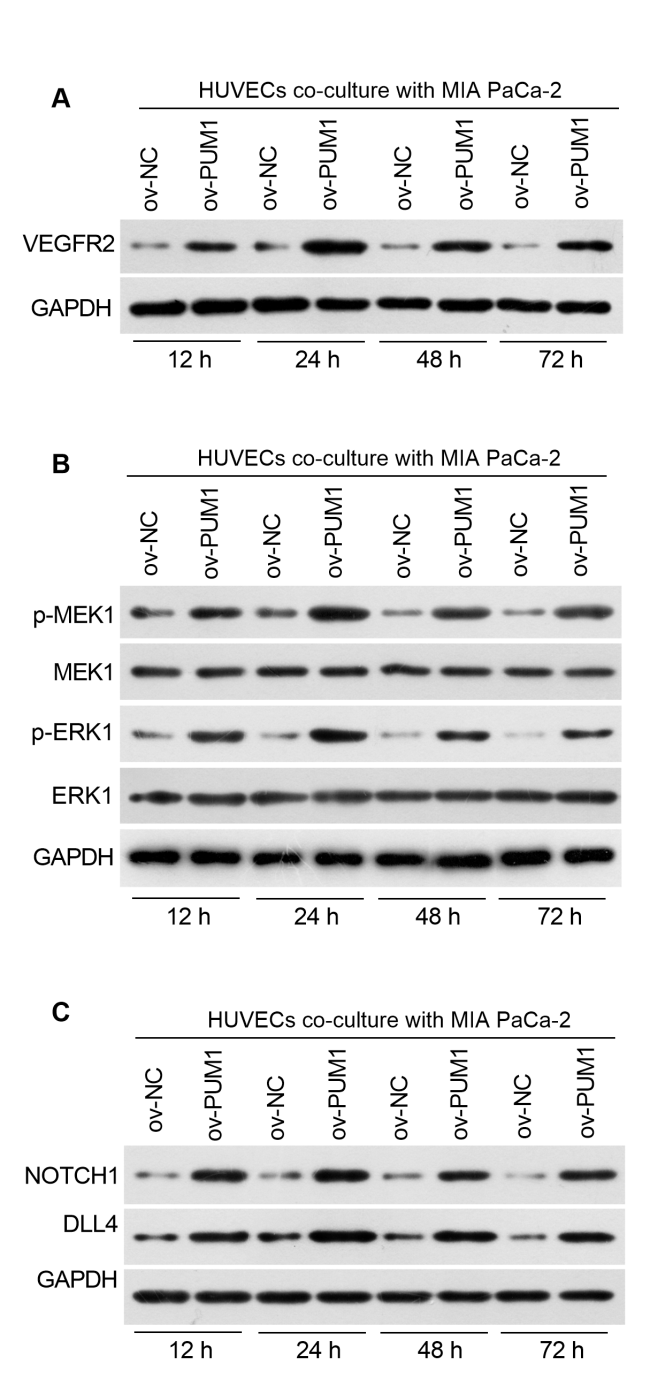
**

Figure S1. Effect of pancreatic cancer cells overexpressed PUM1 on angiogenesis-related signaling of HUVECs at different time-points. To construct a transwell co-culture system, MIA PaCa-2 cells stably overexpressing PUM1 (ov-PUM1) and negative control cells (ov-NC) were cultured in the upper chamber, and HUVECs were cultured in the lower chamber. After co-culture for different time, western blot was performed to detect protein levels.
